# Assessment of cataract surgery outcomes in Nampula (Mozambique): visual acuity, visual function and quality of life

**DOI:** 10.1007/s00417-022-05964-4

**Published:** 2023-01-10

**Authors:** Dulnério Barbosa Sengo, Pires João Saravila, Sancho Sanuel Chivinde, Laura Mavota Mate, Momade Fumo Faquihe, Raul Moragues, Inmaculada López-Izquierdo, Pablo Caballero

**Affiliations:** 1 Faculdade Ciências de Saúde, Universidade Lúrio, Bairro de Marrere, R. Nr. 4250, Km 2,3, Nampula, Mozambique; 2 Departamento de Enfermería Comunitaria, Medicina Preventiva Y Salud Pública E Historia de La Ciencia, Universitat d’Alacant, Carretera Sant Vicent del Raspeig S/N, 03690 Alacante, Sant Vicent del Raspeig Spain; 3 Hospital Central de Nampula, Av Samora Machel, Bairro Central, Nampula, Mozambique; 4 Ministério Dos Combatentes, Av Mártires Machava 307, Maputo, Mozambique; 5 Departamento Estadística Matemáticas E Informática, Universitas Miguel Hernandez, Av de La Universidad S/N 03202, Elche, Spain; 6 Departamento de Física de La Materia Condensada, Universidad de Sevilla, Av. Reina Mercedes S/N 41012, Seville, Spain

**Keywords:** Outcomes, Cataract surgery, Visual acuity, Visual function, Quality of life, Nampula, Mozambique

## Abstract

**Background:**

Despite advances in surgical techniques, cataract remains the leading cause of preventable blindness, and massive surgeries have been adopted as a strategy to change this situation. Monitoring the results of cataract surgeries has become imperative to ensure their quality. Therefore, this study aims to assess the cataract surgery outcomes performed at the Central Hospital of Nampula Mozambique.

**Methods:**

This is a prospective and longitudinal study in which translation, cultural adaptation and validation of the visual function (VF) and quality of life (QoL) questionnaire were performed. The appearance, content, construct, criterion, internal consistency and responsiveness were validated using the most common methods and indicators. Visual acuity (VA), VF and QoL were evaluated on 447 patients before and after surgery by *t*-test and effect sizes.

**Results:**

VF and QoL questionnaires showed one-dimension, good values of TLI (0.973, 0.951) and SRMR (0.057, 0.054), and for each item, weights > 0.7, H2 > 0.5, ranges > 5.8 and the RMSEA < 0.08. Correlations for criterion validity were high and for responsiveness were high for QoL and moderate for VF one and the ordinal Cronbach’s alpha coefficients were greater than 0.97. Difference between VA, VF and QoL before and after surgery was statistically significant (*p* < 0.001). After surgery, 74.3% of patients had good, 23.5% had borderline and 2.2% had poor VA.

**Conclusions:**

The cataract surgery outcomes are outside the WHO recommendations regarding VA, but they have had a great impact on improving VF and QoL. The questionnaires showed excellent psychometric properties and should be used in daily clinical practice to evaluate the results of cataract surgeries. 
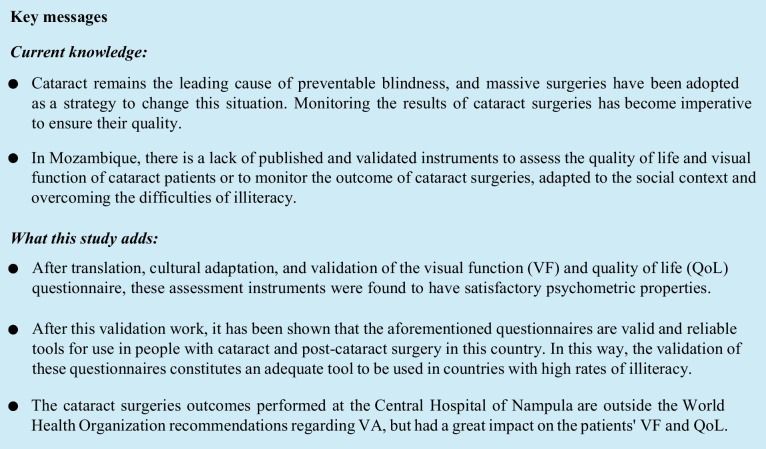

## Introduction

Cataracts are considered the leading cause of preventable blindness in the world and the second leading cause of moderate and severe visual impairment [1]. It is estimated that 10% of the population over 50 years of age has cataract and this prevalence increases to 50% among those aged 65 to 74 years and to 75% for those aged over 75 years [2].

The consequences of vision loss for the individual go far beyond the eye and the visual system; it has been associated with falls, injuries and worsening in domains that encompass mental health, cognition, social function, employment and schooling, affecting independence and quality of life (QoL) [3].

Cataract blindness is recognized as a serious public health problem in developing countries and, therefore, prevention and control programs have been established to reduce its occurrence [4, 5].

In the 5-year action plan for health in Mozambique (2014–2019), regarding ocular health, cataract was defined as one of the priorities, adopting as a strategy the launch of cataract surgery campaigns at a community level [6].

The treatment of cataracts consists, generally, of the surgical removal of the lens and the implantation of an intraocular lens. Despite the advances in techniques for cataract surgery, in many parts of the world, cataracts still prevail as the leading cause of surgically preventable blindness [7].

For a long time, the amount of performed surgeries was emphasized, instead of their results, as an indicator of the performance of cataract surgical services but, fortunately, this is changing, as more emphasis has been placed on the outcome of the surgeries as an indicator, in addition to the number of surgeries performed. In this context, the monitoring of the outcome of cataract services in general, and cataract surgery in particular, has become imperative [8].

Visual acuity (VA) is the most important clinical parameter used to monitor the outcome of surgery, but it is limited with respect to the individual’s ability to perform daily tasks. Clinical measurements quantify the degree of vision loss, not the impact of vision loss [9].

Therefore, in addition to VA, it is important to assess the quality of life and the level of personal satisfaction through questionnaires about the daily tasks of each patient, since studies have shown the importance of evaluating both and achieving acceptable levels of visual outcomes [7, 8].

There is evidence that good results from cataract surgery are an incentive for patients to seek surgical treatment, and vice versa, so it is important to achieve acceptable levels of visual outcome. The World Health Organization (WHO) has suggested the following classification for the visual outcomes of cataract surgery (according to postoperative VA) as “good” from 6/6 to 6/18, “borderline” < 6/18 to 6/60 and “poor” < 6/60, and recommends that, with the correction available, > 80% of the operated eyes should have good visual outcomes (≥ 6/18), < 15% with borderline and only 5% with poor outcomes (< 60/60), and with the best correction is recommended that > 90% of operated eyes have “good” results, < 5% with borderline and < 5% with poor results, up to 6 weeks of follow-up (post-surgery) [8].

Therefore, this study aims to evaluate the results of cataract surgeries performed at the Central Hospital of Nampula (CHN), taking into account visual acuity and sensations related to visual function and quality of life perceived by patients.

## Material and methods

This is a prospective and longitudinal study, carried out in the ophthalmology department of the CHN, which is one of three central hospitals across the country, and the only one in the whole northern region of Mozambique.

This study was divided into two phases, the first phase of translation, cultural adaptation and validation of the VF and QoL questionnaires, and the second phase of evaluating the results of cataract surgery.

### Ethical aspects

This study was previously approved by the Institutional Committee of Bioethics for Health of Lúrio University (CIBSUL), ref: 29.1/Nov/CBISUL/20, and all participants signed an informed consent form to participate in the study.

### Inclusion and exclusion criteria

The patients included in the study were diagnosed with bilateral cataracts, with visual acuity (VA) less or equal than 0.5 (LogMAR) in their best eye and with indication for cataract surgery. All patients under the age of 18 were excluded, as well as those with associated eye diseases (such as retinopathies, maculopathies and uveitis); those with mellitus diabetes or arterial hypertension, previous ophthalmologic surgery or mental problems; and patients who did not return for follow-up after surgery.

### Data collection

First, the VF and QoL questionnaires by Fletcher et al. [10] were contextualized to the Mozambican social context, verifying their feasibility, validity and reliability in patients referred for surgery.

These questionnaires were validated and used in a clinical trial of cataract surgery in India [9, 10], and later used in other studies in countries such as Nigeria [11, 12], Mali [13], Pakistan [14] and China [15, 16], and were considered suitable for use in Pakistan’s national research because they are concise and their administration overcomes the difficulty of illiteracy [14]. This phase took place between October 2019 and March 2020.

Subsequently, the Mozambican versions of the VFm and QoLm questionnaires were applied to patients undergoing cataract surgery before surgery and after cataract surgery (40 days after surgery), and in both moments, in addition to the VF and QoL, the presenting VA was also measured (with correction available, if the participant wears eyeglasses). The surgical technique adopted is the small incision cataract surgery (SICS) with intraocular lens (IOL) implantation, in which a 6-mm incision is made, and at the end of the procedure, there is no need for suturing. The suture is only performed in exceptional cases of occurrence of complications such as rupture of the posterior capsule and loss of the vitreous, for manipulation of the patient. To calculate the intraocular lens, the A-Scan/Pachymeter PacScan 300 Plus ultrasonic biometer was used, and the SKR/T formula was applied. This phase took place between November 2020 and June 2021.

### Procedures

#### Translation, adaptation and validation of VF and QoL questionnaires

The *translation and cultural adaptation (feasibility)* was performed according to the criteria defined by the American Association of Orthopedic Surgeons [17] with the following steps: initial translation, synthesis of translations, retrotranslation, expert committee and pre-test.

For *face and content validity*, an expert panel composed of optometrists resident in Mozambique was consulted to find out whether these adapted questionnaires should include more questions taking into account the realities of Mozambique and, in an affirmative case, a validation process would be performed through the content validity index (CVI) proposed by Lawshe [18].

For *the construct validity*, polychoric correlation matrix was used and the Kaiser–Meyer–Olkin (KMO) test was performed. The quality of the fits between the factorial models and the data was measured using the Tucker-Lewis index (TLI), also known as the non-normed fit index, the root mean square error of approximation (RMSEA) and the standardized root mean squared residual (SRMR). The softer suggested cut-off points recommended for the mentioned indices are TLI values higher than 0.8 and smaller than 0.08 for RMSEA and SRMR. Subsequently, and under the assumptions of the multidimensional item response theory (MIRT), an exploratory analysis was performed to determine the weight of each of the questions on the factors which form the two scales, VFm and QoLm (Table 1). This adjustment of each question was assessed by means of its RMSEA; the variability of the responses to each question was assessed through the factor, known as commonality (H2), the characteristic curve of each question together with its slope, the localization parameters, their range and their information curve, both for each question and the overall scale. Once the number of factors and the weight of each question in each factor were determined, a validation factor analysis was performed.

**Table 1:** Exploratory analysis—result of the parallel analysis for the VFm and QoLm scales, fit indices and eigenvalues of the polychoric matrices

Scale	No. factors according to PA	TLI > 0.8	RMSEA < 0.08	SRMR < 0.08	4 largest eigenvalues			
					1°	2°	3°	4°
VFm	1	0.973	0.100	0.057	9.63	0.79	0.65	0.47
QoLm	1	0.951	0.159	0.054	9.87	0.66	0.37	0.32

*VFm*, adaptation of Fletcher’s Visual Function questionnaire; *QoLm*, adaptation of Fletcher’s Quality of Life questionnaire; *PA*, number of factors according to parallel analysis; *TLI*, Tucker-Lewis index; *RMSEA*, root mean square error of approximation; *SRMR*, standardized root mean square residual

The *criterion validity* was measured using Spearman’s correlation coefficient between both VFm and QoLm, and VA [19]. Even though VA is not the gold standard for VFm or QoLm, presumably, a better VA (lower LogMAR) should be associated with better values for VFm and, therefore, for QoLm. The magnitude of the correlation was determined according to the correlation coefficient, being very high between 0.9 and 1.00, high between 0.7 and 0.9, moderate between 0.5 and 0.7, weak between 0.3 and 0.5 and insignificant between 0 and 0.3 [20].

*Internal consistency* was assessed through Cronbach’s alpha for ordinal coefficients. The value of the coefficient determines the degree of internal consistency of the items in the scale, considered as follows: excellent consistency for values higher than 0.9, good between 0.8 and 0.9, acceptable between 0.7 and 0.8, questionable between 0.6 and 0.7, poor between 0.5 and 0.6 and unacceptable when smaller than 0.5 [21].

*Responsiveness (sensitivity to change)* was assessed using Pearson’s linear correlation between the difference in VA (pre- and post-surgery) and the difference in the VFm and QoLm questionnaires (pre- and post-surgery). The magnitude of the correlation was determined according to the correlation coefficient, being very high between 0.9 and 1.00, high between 0.7 and 0.9, moderate between 0.5 and 0.7, weak between 0.3 and 0.5 and insignificant between 0 and 0.3 [20].

### Assessment of cataract surgery outcomes

Visual acuity (pre- and post-surgery) was measured, monocularly, with available correction (if the patient uses optical correction), using the LogMAR scale.

The results of the cataract surgeries were defined taking into account the VA of the best eye, according to the World Health Organization (WHO) guide for monitoring surgery outcomes, which classifies the outcome as “good” when the VA is better than 0.4 (20/60), “borderline” when VA is between 0.5 and 1.0 (20/60 to 20/200) and “poor” when VA is worse than 1.0 (20/200) [22].

The questionnaires’ final versions (VFm and QoLm) were applied (before and after surgery). The total score for VFm and QoLm was calculated as a percentage, through the total accumulated points divided by the maximum possible score and multiplied by 100. Therefore, a higher score means a better VF and QoL [10].

### Statistical analysis

Means and standard deviation of quantitative variables (age, VA and VFm and QoLm scores) were determined, as well as absolute and relative frequencies of categorical variables (gender, educational level, operated eye).

The *t*-test for paired samples was performed to compare VA, VF and QoLm before and after surgery. A two-sided *p* value < 0.05 was considered to be statistically significant. The effect of surgery on VA, VF and QoL W was estimated by Cohen’s delta (Δ), being considered weak for Δ ≤ 0.1, medium for Δ > 0.1 and ≤ 0.3 and strong for Δ ≥ 0.5 [23]. All data were analysed using IBM SPSS statistics version 22.0.

## Results

### Validation of the VFm and QoLm questionnaires versions

After the translation and cultural adaptation to the Mozambican context of the VF and QoL questionnaires, the psychometric properties of the final versions VFm and QoLm were evaluated.

#### Face and content validity

There was no suggestion of changes or inclusion of more questions by the experts consulted, and there were no new aspects related to VF and QL that should be included to adapt the questionnaires (VFm and QoLm) to the cultural context of Mozambique (the final VFm and QoLm can be found in Tables 6 and 7 in Annex 1 of this study).

#### Construct validity

The KMO test returned a value of 0.93 for the VFm scale and 0.91 for the QoLm scale, both well over 0.8, so the existence of underlying factors is clear in both scales.

The parallel analysis showed the necessity for a single underlying component or dimension in both VFm and QoLm. The classical criterion of number of eigenvalues larger than 1 also showed no necessity for more than one factor.

The SRMR fit indices are within the limits, but the TLI and RMSEA are slightly over the desirable thresholds. Only the first value in both cases exceeds 1, whereas the second value is very small in both scales.

Under the assumptions of MIRT, Table 2 presents the results of each of the questions that compose both scales under the assumption of a single factor. It should be noted that the weights are between 0.723 and 0.955 in the case of the VFm scale and between 0.798 and 0.960 for the QoLm scale.

**Table 2:** Characteristics of the questions that compose the unidimensional VFm and QoLm scales

Question	$\overline{X }$	sd	Weight	H2	Slope	Coefficients				RMSEA < 0.08
					A	B1	B2	B3	Range	
VFm										
1	0.478	0.603	0.723	0.522	1.780	− 0.459	− 4.368	− 6.549	8.329	0.059
2	0.635	0.749	0.858	0.736	2.841	− 0.165	− 3.617	− 8.504	11.345	< 0.001
3	0.393	0.657	0.749	0.561	1.924	− 1.259	− 3.911	− 6.215	8.139	< 0.001
4	1.27	1.044	0.900	0.811	3.522	2.427	− 1.258	− 3.889	7.411	< 0.001
5	0.702	0.912	0.855	0.731	2.803	− 0.290	− 2.852	− 5.369	8.172	0.035
6	1.011	1.145	0.898	0.807	3.483	0.565	− 2.169	− 3.651	7.134	0.036
7a	0.865	0.959	0.838	0.702	2.610	0.431	− 2.288	− 4.643	7.253	0.035
7b	0.815	0.905	0.776	0.603	2.096	0.333	− 2.145	− 4.529	6.625	0.046
8	1.365	1.113	0.955	0.911	5.456	3.297	− 0.741	− 4.670	10.126	< 0.001
9	1.601	1.142	0.921	0.849	4.033	3.468	0.211	− 2.094	6.127	< 0.001
10	1.685	1.131	0.920	0.846	3.994	3.705	0.649	− 1.855	5.849	0.057
11a	1.062	0.903	0.907	0.823	3.666	1.962	− 1.840	− 6.650	10.316	0.036
11b	0.933	0.936	0.794	0.631	2.224	0.843	− 1.779	− 4.039	6.263	0.056
Total	0.986	0.762	Total % ($\overline{X }(sd)$)			33.0%	(25.4%)			
QOLm										
1	2.017	1.122	0.945	0.893	4.915	5.35	2.392	0.068	5.282	< 0.001
2	2.039	0.994	0.909	0.827	3.723	5.054	2.588	− 0.892	5.946	0.049
3	2.118	1.043	0.953	0.908	5.356	7.036	3.28	0.269	6.767	0.043
4	2.169	1.044	0.945	0.894	4.934	6.623	2.979	0.682	5.941	< 0.001
5	1.719	1.155	0.949	0.900	5.101	4.035	1.118	− 1.912	5.947	0.031
6	1.764	1.11	0.948	0.898	5.056	4.509	1.713	− 2.109	6.618	0.03
7	1.528	1.1	0.96	0.922	5.847	4.294	0.417	− 3.936	8.23	< 0.001
8	1.596	1.033	0.927	0.860	4.219	4.082	0.596	− 3.189	7.271	0.034
9	1.618	1.025	0.945	0.894	4.936	4.651	1.024	− 3.71	8.361	< 0.001
10	1.697	1.073	0.846	0.715	2.699	2.812	0.677	− 1.767	4.579	0.014
11	1.197	0.939	0.798	0.637	2.256	1.843	− 0.934	− 3.659	5.502	< 0.001
12	1.663	1.041	0.869	0.755	2.986	3.000	0.942	− 2.236	5.236	< 0.001
Total	1.76	0.909	Total % ($\overline{X }(sd)$)			58.7%	(30.3%)			

*H2*, commonality; *RMSEA*, root mean square error of approximation

The commonalities or percentage of explained variance of the variable by the factor is always higher than 50% for VFm scale and 60% in the case of the QoLm scale. Regarding the slopes, they take values between 1.780 and 5.456 in VFm, whereas in the QoLm scale, their values are between 2.256 and 5.847. Regarding the range denoted by B1–B3, the values oscillate between 5.849 and 11.345 for the VFm scale and between 4.579 and 8.361 for QoLm. The values of RMSEA are lower than 0.06 in all cases.

In the confirmatory analysis (Table 3), the TLI, RMSEA and SRMR values are within the intervals established as a good fit except for the RMSEA value which resulted one hundredth over the threshold.

**Table 3:** Results of confirmatory analysis for the VFm and QoLm, fit indices

Scale	Number of factors	TLI > 0.8	RMSEA < 0.08	SRMR < 0.08
VFm	1	1.004	< 0.001	0.046
QoLm	1	0.998	0.090	0.057

*VFm*, adaptation of Fletcher’s questionnaire for Visual Function; *QoLm*, adaptation of Fletcher’s Quality of Life questionnaire; *TLI*, Tucker-Lewis index; *RMSEA*, root mean square error of approximation; *SRMR*, standardized root mean square residual

#### Criterion validity

There was a high correlation between VA and VFm before surgery (Rs =  − 0.8) and after surgery (Rs =  − 0.89), with negative values in both cases. As for VA and QoLm, there was similarly a statistically significant correlation before (Rs =  − 0.88) and after (Rs =  − 0.84) the surgery, also negative in both cases.

#### Reliability of VFm and QoLm

##### Internal consistency

Cronbach’s ordinal alpha coefficients for the polychoric correlation matrices of VFm and QoLm were 0.97 (IC 95%: 0.96; 0.98) and 0.98 (IC 95%: 0.96; 0.98) and over the threshold of 0.95.

##### Responsiveness

**Fig. 1:**
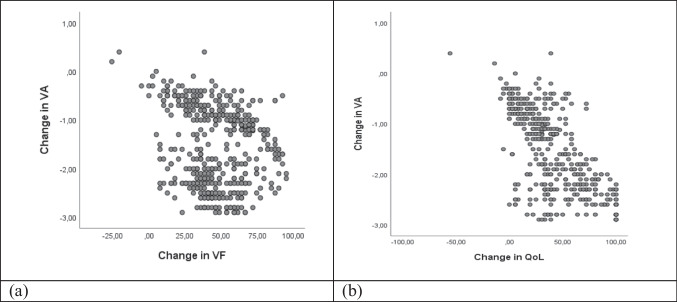
Scatterplot and regression line between VA and **a** variation in the score of the VFm questionnaire and **b** variation in the score of the QoLm questionnaire

Figure 1 shows how the variation in VA (pre- and post-surgery) correlates with the variation in both VFm and QoLm questionnaires (pre- and post-surgery). In both cases, the correlation was significant, with moderate values (0.63) for VFm and high values (0.84) for QoLm.

### Assessment of cataract surgery outcomes

Of the 484 patients operated on during the study period, 447 (92.4%) attended the follow-up until the 40th day after cataract surgery and, therefore, were part of the study, and of these, only 5 (1.1%) required suturing in the wound.

Patients were aged between 30 and 92 years (with a mean of 66.38, SD 11.06). Most patients were male (54.8%), illiterate (53.2%) and operated on both eyes (70.2%) (Table 4). Before surgery, almost 3 out of 4 patients had poor VA (72.3%), and after surgery, almost 3 out of 4 patients had good VA (74.0%) and only 2.2% had a poor outcome after surgery. The results of the surgery were statistically significant (*p* < 0.001) and strong effects between pre- and post-surgery. Regarding VF and QoL, the results are similar with highly significant effect sizes.

**Table 4:** Socio-demographic characteristics associated with cataract surgery on VA, VFm and QoLm outcomes

	N (%)	Change of means (effect size) *Sig*			Post-surgery *N* (%)		
		VA	VF	QoL	Good	Borderline	Poor
Total	447 (100%)	− 1.5 (1.8) < *0.01*	46 (2.2) < *0.01*	40 (1.3) < *0.01*	331 (74.0)	106 (23.7)	10 (2.2)
Age (years)							
30–49	40 (8.9)	− 1.6 (1.6) < *0.01*	42 (2.1) < *0.01*	41 (1.3) < *0.01*	33 (82.5)	7 (17.5)	0 (0.0)
50–69	228 (51.0)	− 1.4 (1.7) < *0.01*	46 (2.1) < *0.01*	35 (1.2) < *0.01*	179 (78.5)	46 (20.2)	3 (1.3)
≥ 70	179 (40.0)	− 1.7 (1.7) < *0.01*	47 (2.1) < *0.01*	48 (1.2) < *0.01*	119 (66.5)	53 (29.6)	7 (3.9)
Gender							
Male	245 (54.8)	− 1.8 (1.7) < *0.01*	45 (2.3) < *0.01*	40 (1.3) < *0.01*	182 (74.3)	59 (24.1)	4 (1.6)
Female	202 (45.2)	− 1.5 (1.8) < *0.01*	48 (2.2) < *0.01*	41 (1.3) < *0.01*	149 (73.8)	47 (23.3)	6 (3.0)
Educational level							
Illiterate	238 (53.2)	− 1.6 (1.8) < *0.01*	46 (2.4) < *0.01*	44 (1.4) < *0.01*	172 (72.3)	60 (25.2)	6 (2.5)
Primary	146 (32.7)	− 1.5 (1.8) < *0.01*	45 (2.0) < *0.01*	38 (1.2) < *0.01*	102 (69.9)	40 (27.4)	4 (2.7)
Secondary	55 (12.3)	− 1.3 (1.7) < *0.01*	50 (2.1) < *0.01*	32 (1.2) < *0.01*	49 (89.1)	6 (10.9)	0 (0.0)
College	8 (1.8)	− 2.1 (2.3) < *0.01*	47 (7.7) < *0.01*	39 (2.7) < *0.01*	8 (100.0)	0 (0.0)	0 (0.0)
Operated eyes							
Only one	133 (29.8)	− 1.5 (1.8) < *0.01*	43 (2.2) < *0.01*	38 (1.2) < *0.01*	79 (59.4)	48 (36.1)	6 (4.5)
Both	314 (70.2)	− 1.8 (1.8) < *0.01*	47 (2.2) < *0.01*	42 (1.3) < *0.01*	252 (80.3)	58 (18.5)	4 (1.3)

Before surgery, the general, visual perception and peripheral vision subscales were the most affected (with the lowest scores) on the VFm scale, while for the QoLm scale, the mental, mobility and social subscales were most affected. Surgery had a strong effect (Δ > 0.5) on VF and QoL, with the greatest effect on the visual perception and mental subscales, respectively. There was a statistically significant difference between pre- and post-surgery VFm and QoLm scores (Table 5).

**Table 5:** VFm and QoLm scores and comparison between pre- and post-surgery scores

Subscales	Pre-surgery	Post-surgery	Mean difference (SD)	Effect size	*p*
	Mean (SD)	Mean (SD)			
**Visual function**					
General	20.3 (20.6)	79.9 (24.8)	59.5 (28.7)	2.9	< 0.001
Visual perception	26.4 (23.3)	81.0 (18.6)	54.6 (25.2)	2.3	< 0.001
Peripheral vision	27.6 (32.2)	82.0 (22.5)	54.5 (31.8)	1.7	< 0.001
Sensory adaptation	40.0 (22.0)	76.8 (16.8)	36.8 (24.0)	1.7	< 0.001
Depth perception	40.9 (35.4)	86.1 (21.1)	45.2 (34.1)	1.3	< 0.001
Total	33.5 (20.6)	79.5 (16.8)	46.0 (21.3)	2.2	< 0.001
**Quality of life**					
Self-care	59.3 (34.8)	93.8 (15.9)	34.6 (33.9)	2.7	< 0.001
Mobility	46.2 (34.0)	88.4 (19.6)	42.2 (32.7)	2.6	< 0.001
Social	46.9 (32.9)	90.8 (18.4)	43.9 (32.0)	2.8	< 0.001
Mental	45.5 (31.0)	90.3 (18.9)	44.8 (30.5)	2.9	< 0.001
**Total**	50.5 (31.4)	91.1 (16.9)	40.6 (29.8)	2.9	< 0.001

**Fig. 2:**
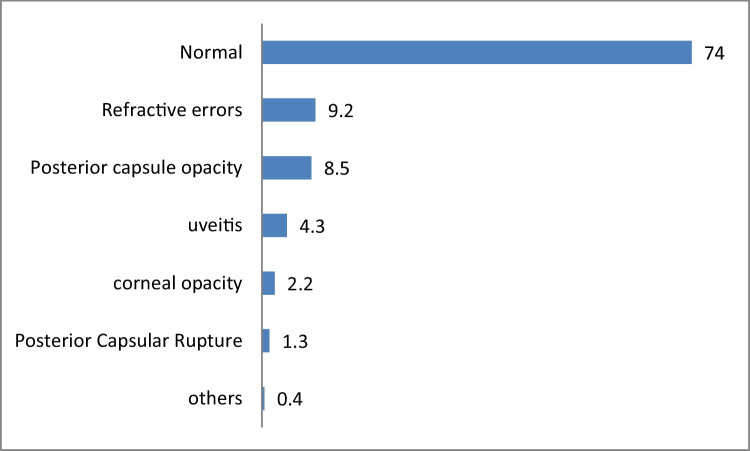
Causes of visual impairment after cataract surgery

According to Fig. 2, the major cause of visual impairment after surgery was uncorrected refractive errors and posterior capsule opacity.

## Discussion

Cataracts are considered the leading cause of avoidable blindness in the world [1], and, in order to combat it, Mozambique has adopted a strategy of the implementation of campaigns of cataract surgery in communities [6]. However, on the other hand, there is a need to ensure better follow-up and quality monitoring of these surgeries.

The assessment of cataract surgery outcomes taking into account VA, VF and QoL is a pioneering initiative in Mozambique, given the scarcity of published studies on cataract surgery outcomes and their impact on patients’ VF and QoL.

### Validation of the VFm and QoLm questionnaires

The translated and adapted questionnaires (VFm and QoLm) showed good psychometric characteristics. During this process, parallel analysis suggested that both questionnaires are unidimensional in this context. The fit indices for both the data used in the exploratory and confirmatory analysis show the fit to the unidimensional model. The fit indices were optimal, and despite some not being within the optimal limits, they were very close to them.

The detailed study of the items in each questionnaire showed that every item in both questionnaires has a weight higher than 0.7, greatly over the 0.3 recommended value to be able to state that the item in question contributes relevant information about the latent factor that one intends to measure. With this information, we discarded eliminating any item using this criterion.

It is assumed that good visual acuity will provide the patient with better visual function and, hence, better quality of life. Therefore, a tool that measures VF or QoL should be correlated with VA. In this case, both questionnaires have a high correlation with VA, both before and after surgery, which confirms the validity of the used criterion.

Additionally, as showed by Cronbach’s ordinal coefficients, the questionnaires are endowed with internal consistency. Regarding the responsiveness of the questionnaires, it is observed that after the cataract surgery, it was possible to alter the VA in higher or lower degree, with this effect of the alteration of VA being highly correlated with the change in VF, but much more correlated still with the variations in QoL.

### Assessment of cataract surgeries according to the VA, VFm and QoLm questionnaires

Before surgery, most participants had poor VA and consequently a low VF and QoL score, given the significant relation between VA and VFm and QoLm scores. Before surgery, the general, visual perception and peripheral vision subscales were the most affected in VF, corroborating the results found by Fletcher et al. [10], while for Zhou et al. [15] in addition to the general subscale, depth perception and sensory adaptation were more affected. Regarding QoL, the mental, mobility and social subscales were more affected, and similar results were found by Fletcher et al. [10], whose mobility and social were more affected, and by Zhou et al. [15], whose mental and mobility scores were lower, which demonstrates the impact of cataract, especially in the mobility aspect.

After surgery, in general, the majority (74.3%) of the patients had a “good” VA with the available corrections, and a minority with “borderline” and “poor” (23.5% and 2.2%, respectively), being these results close to those recommended by the World Health Organization (according to which, at least 80% should have a “good” result, and no more than 15% and 5% should have result “borderline” and “poor” respectively) [22]. The main causes of borderline and poor VA after surgery were uncorrected refractive errors and posterior capsule opacity. Therefore, comparing with results found in studies carried out in Nepal [24], Bangladesh [25] and Nigeria [26], our study had better results. In Nigeria [26], the main causes of poor vision after surgery were uncorrected refractive errors and posterior capsule rupture (7.0%).

Refractive errors stand out among the main causes of visual impairment after cataract surgery. Studies performed in Mozambique have shown that the acquisition of corrective glasses is still not easily accessible for a large proportion of the population of Nampula, with financial difficulties being indicated as the main barrier, in a country where 49.9% of the population lives in conditions of severe poverty [27, 28], resulting in the circumstance that some people who undergo surgery can continue having visual deficiencies due to uncorrected refractive errors. Therefore, for our study, the presenting visual acuity (with the available correction) was used as a reference over visual acuity with better correction since it better reflects the daily reality of the patient, being more associated to the characteristics that wish to be measured such as VFm and QoLm.

Regarding VF and QoL, there was a significant improvement after surgery, and the effect of surgery was greater in the perception and mental subscales, respectively. These results coincide with those found by Zhou et al. [15] as well as Fletcher et al. [10], showing that cataract surgery effectively improves VA and, consequently, patients’ VF and QoL.

We are talking about making it easier to carry out day-to-day activities, as well as recognizing people and small objects. On the other hand, a better VA improves the patient’s mood and increases confidence to perform daily activities’ therefore, the psychosocial impact of surgeries is undeniable, representing a differential between pre- and post-surgical.

Although surgery has a greater impact on the VF scale, as VF addresses aspects directly related to vision, QoL focuses on broader aspects that may be influenced by vision. We have seen that cataract surgery does not only impact vision, but health as a whole, since according to the World Health Organization (WHO), health is a complete physical, mental and social well-being, and not just absence of illness [29].

The results of this study show that some patients undergo cataract surgery when aspects of their VF and QoL are already very debilitated, compromising the psychosocial well-being of the individual and his family. Therefore, these instruments (VFm and QoLm) may also help in the management and decision making by clinicians, such as, for example, the choice of the best time to operate, since studies have revealed that one of the barriers of access to cataract surgery has been the fact that “the cataract is not yet mature enough” [28, 30–32] leading the doctor or the patient to choose to wait for the appropriate time to perform the surgery.

These results are not bad considering the results of other studies, but they are outside the recommended by the WHO. There is a need for future studies to understand the magnitude of residual refractive error after surgery and associated factors, as well as experimental studies between different therapies seeking better solutions for the occurrence of complications after surgery, especially posterior capsule opacities.

This study was limited with respect to the relatively small sample size, due to the reduction in the number of cataract surgeries at the Central Hospital of Nampula during the COVID-19 pandemic. On the other hand, a longer follow-up would allow better monitoring of results.

The use of instruments that assess subjective aspects related to the patient’s daily life to monitor the results of surgeries is a milestone and inflection point for eye health care in Mozambique, since these instruments provide important complementary information, enabling the clinician to better understand the real impact of the cataract as well as the surgery itself on the patient’s daily life, and thus be able to provide more personalized health care, conveying greater confidence and security to the patient. Therefore, the introduction of VF and QoL assessment instruments in routine ophthalmology examinations is suggestive.

## Conclusion

Therefore, through this study, VF and QoL assessment instruments with satisfactory psychometric properties were obtained, being valid and reliable options for use in people with cataracts and after cataract surgery.

Patients undergo cataract surgery when certain aspects of their VF and QoL are already compromised, and the results of surgeries performed at the HCN still do not comply with the WHO recommendations regarding VA, but have a great impact on the VF and QoL of patients. The application of questionnaires that assess the patient’s daily difficulties in tasks associated with vision is an asset, given the broader perception they offer about the patient’s health status, therefore a challenge for health managers, as well as for eye health professionals in Mozambique.

## Annex 1 Original versions of the VF and QoL questionnaires by Fletcher et al. [10]

Table 6

**Table 6:** Vision Function questionnaire

Question no	Question	Rating			
		Not at all	A little	Quite a lot	A lot
1	In general, would you say your vision (with glasses if you wear them) is:	1(very good)	2 (good)	3 (fair)	4 (poor)
2	To what extent does your sight limit you in your daily activities?	1	2	3	4
3	How much problem do you have recognizing people across the street?	1	2	3	4
4	How much problem do you have recognizing the face of a person standing near you?	1	2	3	4
5	How much problem do you have recognizing small or minute objects (such as grains or the lines in your hand)?	1	2	3	4
6	When you are walking along, how much problem do you have noticing objects off to the side?	1	2	3	4
7a	How much problem do you have adjusting to darkness after being in bright light?	1	2	3	4
7b	How much problem do you have adjusting to brightness after being in a dark place?	1	2	3	4
8	How much problem do you have locating something when it is surrounded by a lot of other things (like finding a specific food item on your plate)?	1	2	3	4
9	How much problem do you have in recognizing colours?	1	2	3	4
10	When you reach for an object (e.g., to take a glass), how much problem do you have in finding it, because it is further away or closer than you thought?	1	2	3	4
11a	How much problem do you have in recognizing a person when you are in bright light?	1	2	3	4
11b	How much problem do you have seeing with bright lights shining on your eyes (such as from an oncoming bus or car)?	1	2	3	4

The Portuguese version of the VF questionnaire is available from the corresponding author on reasonable request

Table 7

**Table 7:** Quality of Life questionnaire

**Activity**	Rating			
	Not at all	A little	Quite a bit	A lot
**Self-care**				
How much problem do you have because of your vision in doing the following activities unaided?				
Bathing	1	2	3	4
Eating	1	2	3	4
Dressing	1	2	3	4
Toileting	1	2	3	4
**Mobility**				
How much problem do you have because of your vision in doing the following activities unaided?				
Walking to neighbours	1	2	3	4
Walking to shops	1	2	3	4
Doing your usual household chores	1	2	3	4
**Social**				
Because of your visual problems, do you feel less inclined to participate in the following?				
Attending social functions like weddings, funerals, festivals	1	2	3	4
Meeting with friends and relatives	1	2	3	4
**Mental**				
Because of your vision problems do you feel:				
A burden on others	1	2	3	4
Dejected	1	2	3	4
Loss of confidence in doing usual activities	1	2	3	4

The Portuguese version of the QoL questionnaire is available from the corresponding author on reasonable request
